# Platelets in the tumor microenvironment and their biological effects on cancer hallmarks

**DOI:** 10.3389/fonc.2023.1121401

**Published:** 2023-03-03

**Authors:** Lilan Chen, Chunyan Zhu, Fan Pan, Ying Chen, Lei Xiong, Yan Li, Xiaoyuan Chu, Guichun Huang

**Affiliations:** ^1^ Department of Medical Oncology, Affiliated Jinling Hospital, Medical School of Nanjing University, Nanjing, Jiangsu, China; ^2^ Division of Immunology, Medical School of Nanjing University, Nanjing, Jiangsu, China; ^3^ Department of Cardio-Thoracic Surgery, Affiliated Jinling Hospital, Medical School of Nanjing University, Nanjing, Jiangsu, China; ^4^ Department of Respiratory Medicine, Affiliated Drum Tower Hospital, Medical School of Nanjing University, Nanjing, Jiangsu, China

**Keywords:** platelets, cancer biology, tumor microenvironment, tumor-associated platelets, tumor metastasis

## Abstract

The interplay between platelets and tumors has long been studied. It has been widely accepted that platelets could promote tumor metastasis. However, the precise interactions between platelets and tumor cells have not been thoroughly investigated. Although platelets may play complex roles in multiple steps of tumor development, most studies focus on the platelets in the circulation of tumor patients. Platelets in the primary tumor microenvironment, in addition to platelets in the circulation during tumor cell dissemination, have recently been studied. Their effects on tumor biology are gradually figured out. According to updated cancer hallmarks, we reviewed the biological effects of platelets on tumors, including regulating tumor proliferation and growth, promoting cancer invasion and metastasis, inducing vasculature, avoiding immune destruction, and mediating tumor metabolism and inflammation.

## 1 Introduction

As early as 1865, an association between cancer and thrombosis, closely linked to platelets, was observed by Trousseau (1). Clinical evidence suggested that thrombocytosis (elevated platelet counts) was correlated with increased cancer risk (2, 3), and high platelet-to-lymphocyte ratio (PLR) or increased platelet count was revealed as an adverse prognostic factor (4, 5). Technological advancement has made it possible to analyze proteins and RNAs of platelets comprehensively, challenging the old understanding of platelet transcriptome (6, 7). As a result, numerous studies have revealed that the proteins and RNAs within platelets vary among individuals with or without cancer, as well as between different cancer types. Tumor-educated platelets (TEPs), which are platelets isolated from the circulation of cancer patients and have distinct RNA and protein profiles, have emerged as potential indicators in liquid biopsies (8).

Platelets play a crucial role in invasive potential, intravasation, and survival in the circulation, arrest, adhesion, and extravasation into secondary sites during the hematogenous spread of tumor cells (9, 10). Platelets probably affect other aspects of cancer other than hematogenous metastasis. The hallmarks of cancer have evolved in 2022 as a result of further understanding of cancer (11). The initial six hallmarks of cancer include sustaining proliferative signaling, evading growth suppressors, resisting cell death, enabling replicative immortality, inducing/accessing vasculature, and activating invasion and metastasis (12). Two more “emerging hallmarks” comprise reprogramming cellular metabolism and avoiding immune destruction (13). Unlike the aforementioned acquired capabilities, genome instability and tumor-promoting inflammation were defined as “enabling characteristics”. With increasing recognition of the tumor microenvironment (TME) in cancer development and the rapid progress of both the breadth and depth of cancer research, four new concepts comprising unlocking phenotypic plasticity, non-mutational epigenetic reprogramming, polymorphic microbiomes, and senescent cells were put forward (11). Platelets might play complex roles in multiple steps of tumor development and affects several hallmarks of cancer.

The tumor microenvironment is a complicated system composed of various non-cancerous cells (e.g., endothelial cells, fibroblasts, adipocytes, and immune cells) and non-cellular components (e.g., extracellular matrix, cytokines, growth factors, and extracellular vesicles). Platelets were engaged in tumor development along with other components of the tumor microenvironment through abundant tumor neovascularization (14). Evidence showed that platelets probably existed in the TME, but the underlying molecular mechanisms remain to be explored (15). We speculated that except the direct contact with tumor cells in the circulation, platelets could also adhere to highly permeable blood vessels and extravasate into tumor stroma through vascular leakage or other molecular pathways.

**Figure 1:**
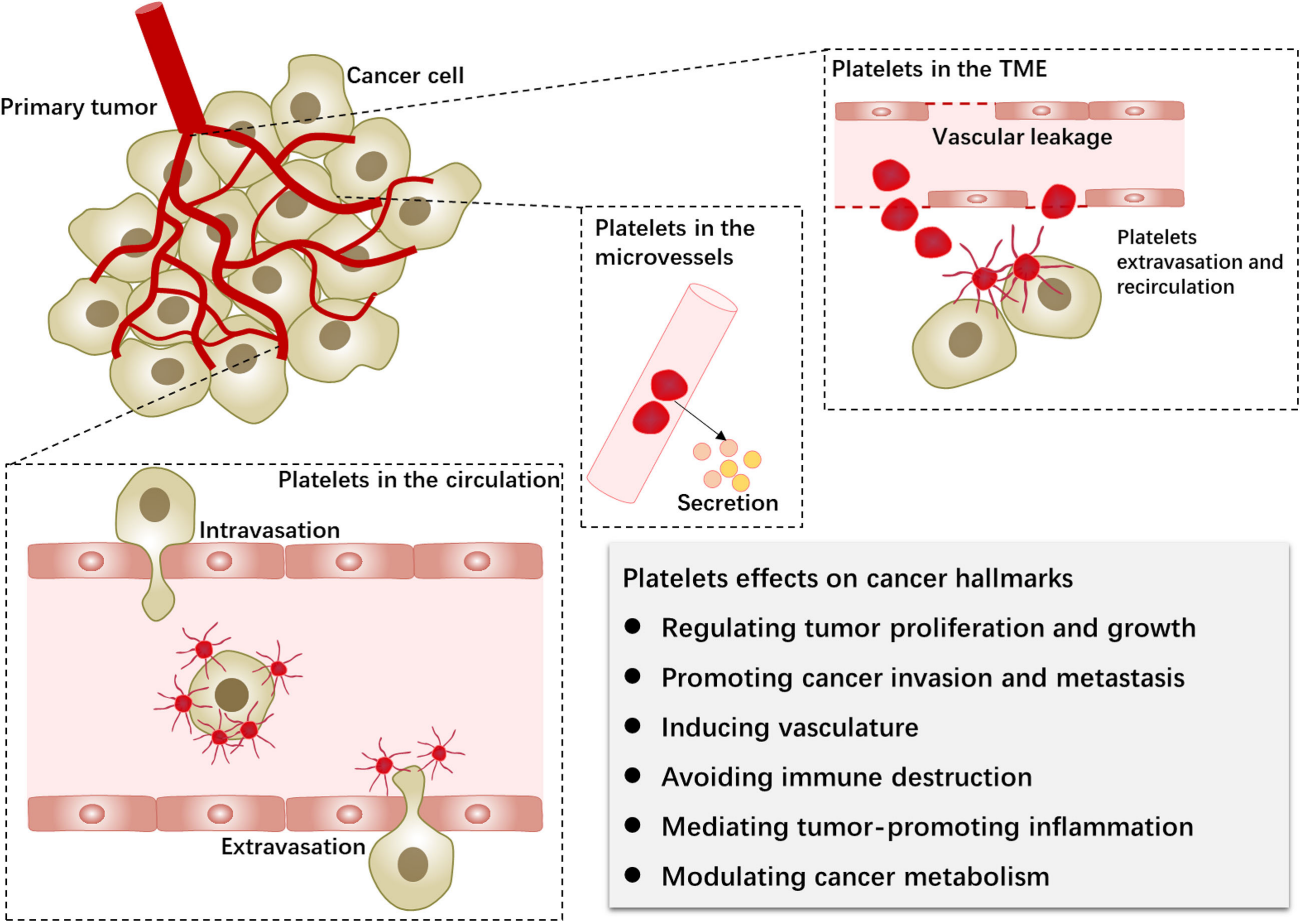
Platelets in the primary tumor microenvironment and hematogenous metastasis. Platelets and tumors interact during the hematogenous dissemination of tumor cells. Furthermore, platelets extravasate into the tumor microenvironment via neovascularization leakage. Platelet-tumor cell interactions can affect the biological behavior of tumor cells (cancer hallmarks) through platelet activation, surface receptors, and released factors.

As a particular cellular component, the role of platelets in the TME has not been fully revealed, and the specific contributions of platelets to cancer require constant exploration. Thus, in conjunction with advances in cancer concepts, this review provides a synopsis of the contributions of platelets to cancer progression to provide a clear map for future research (**Figure 1**).

## 2 Platelets in the tumor microenvironment

Recent studies associated with cancer biology no longer focused solely on tumor cells but were based on the network of TME. To better understand the biological significance of platelets in cancer progression, the precise locations of platelet–tumor interactions need to be figured out. Research found that platelets infiltrated into the tumor stroma and had the predictive value in patients with pancreatic and colorectal cancer (16–18). Platelets exist in the TME of ovarian, melanoma, lung, and colorectal cancers (15, 19–22). Through the injection of platelets labeled with yellow fluorescence protein (YFP), extravascular platelets were found in the tumor bed, and the platelets outside blood vessels were tumor-specific compared with those in the peritonitis (20, 22). Therefore, like tumor-associated fibroblasts, platelets in the primary tumor microenvironment could be termed tumor-associated platelets (TAPs).

Not only platelet secretion but also the platelet itself can regulate the network of TME through vascular leakage (14). Platelet depletion by anti-platelet antibodies can significantly reduce microvessel density (20). Moreover, platelets in the primary TME can modulate the structure of tumor vessels by inducing vascular permeability (21). Platelets participate in vascular maturation and change the extracellular matrix (ECM), which is connected to the capacity of tumor cells to extravasate from the original site, indicating that platelet strongly affects the initial invasion of tumor cells. Meanwhile, the process of platelet extravasation has been explored. Platelet focal adhesion kinase (FAK) protein regulates their migration into TME (22).

Platelets play multiple roles in cancer biology through platelet-related molecules in the tumor microenvironment. The endocytic mechanism of platelets can ingest and store proteins derived from a tumor, thereby modulating the tumor microenvironment (23). Otherwise, platelet-derived microparticles (PMPs) infiltrating solid tumors can transfer RNAs to tumor cells (24). Micro-RNAs (miRNAs) and microparticles derived from platelets have emerged as novel research targets. Additionally, the bone and bone marrow microenvironment, which are connected with cancer bone metastasis, can facilitate the communication between cancer cells and platelets (25, 26).

### 2.1 Effects of TAPs on tumor proliferation and growth

Tumor cells have the fundamental hallmarks of sustaining proliferative signaling, evading growth suppressors, and resisting cell death. As has been demonstrated, platelets promote tumor growth and proliferation in ovarian, breast, lung, glioma, and hepatocellular carcinoma, as well as osteosarcoma (27–32). Activation of platelets was reported to contribute to tumor growth *in vivo* in pancreatic cancer (33). After the anti-angiogenic therapy withdrawal, the platelet infiltration in TME increases, and the tumor growth accelerates (22). However, another study observed that the interactions between platelets and tumor cells in colorectal cancer led to the release of different types of microparticles which, although inducing epithelial-to-mesenchymal transition of tumor cells, incurred the intratumoral macrophages and reduced tumor growth (15).

Additionally, the glycoprotein (GP)VI on the platelet surface aided in the growth of primary tumors in Lewis lung carcinoma or melanoma (34). The latest study using a mouse model of hepatocellular carcinoma (HCC) with non-alcoholic fatty liver disease (NAFLD) also found an unexpected result that platelets suppressed liver cancer growth *via* activating CD8^+^T cells (35). Although most studies have suggested that platelets facilitate tumor growth, the precise mechanisms behind these phenomena remain unknown. It appears that different tumor types and different platelet-derived receptors and secretions had distinct effects on tumor proliferation and growth.

### 2.2 Effects of TAPs on cancer invasion and metastasis

The effects of tumor cells-TAPs interaction on invasion-metastasis cascades have been confirmed, including promoting epithelial-mesenchymal transition (EMT) of cancer cells, protecting circulating tumor cells (CTCs) survival in the bloodstream, enhancing adhesion to endothelial cells, and further extravasating into distant sites (15). Growing evidence suggests that anti-platelet agents are effective in hindering tumor metastasis. Upon tumor cells detaching from the primary tumor, invading the surrounding site, and subsequently arriving in the bloodstream, platelets are the first cells to contact with. Tumor cells initiate platelet activation through various mechanisms for stable adhesion, which allows platelets to protect CTCs from high shear stress and immune surveillance (36). Coating with platelets, tumor cells have increased adhesion to the vessel wall and be trapped within capillaries. The secretion of bioactive molecules from platelets assists tumor cells in vascular permeability and trans-endothelial migration. Several molecular mechanisms have been reported in this process, including Fcγ receptor IIa (FcγRIIa), C-type lectin-like receptor 2 (CLEC-2), glycoprotein VI (GPVI), transforming growth factor-β (TGF-β), heat shock protein 47 (HSP47), integrin α2β1& α6β1, adenosine triphosphate (ATP), and lysophosphatidic acid (LPA).

The immunoreceptor tyrosine-based activation motif (ITAM) containing receptors on platelets were rarely studied on the cross-talk between platelets and tumor cells. FcγRIIa, CLEC-2, and GPVI have been investigated as key members of these receptors for mechanisms concerning thrombosis and metastasis. FcγRIIa is required for prostate tumor cell-induced platelet activation (TCIPA), which is dependent on adenosine 5′-diphosphate (ADP) secretion from dense platelet granules, followed by platelet aggregation (37). Downstream signals of the ITAM cascade are potential mediators of tumor cell-induced platelet secretion (TCIPS), such as Syk kinase, phospholipase C, and protein kinase C. Pharmacologic antagonists of these signals as well as inhibitors of FcγRIIa have a critical impact on TCIPS and TCIPA. It was assumed that when platelets are exposed to prostate cancer cells, integrin αIIbβ3-FcγRIIa-P2Y12 cross-talk transduces the signal to release ADP whose receptor P2Y12 amplifies the response, thereby promoting platelet aggregation. Previously, the collaborative signaling of integrin αIIbβ3 and FcγRIIa was established during thrombus formation (38). Furthermore, immunoglobulin G (IgG) derived from cancer cells acts on platelet FcγRIIa, leading to platelet activation (39). Taken together, integrin/ITAM pair, cancer cell derived-IgG, FcγRIIa on platelets, and ADP secretion may be potential targets to cut off signaling during cancer cell induced-platelet activation.

CLEC-2, primarily expressed on platelets, is a receptor for platelet-activating snake venom and contains a hemi-ITAM (40). Podoplanin (PDPN), also known as aggrus, encodes a glycoprotein associated with cell migration and adhesion and is over-expressed in a variety of tumor cells (41). As the key endogenous ligand for CLEC-2, PDPN plays crucial roles in inducing platelet activation and aggregation (42). The research showed that thrombus formation was suppressed and experimental hematogenous metastasis in lungs was decreased in CLEC-2-depleted mice (43). Moreover, recombinant rhodocytin was generated by binding to CLEC-2 to inhibit platelet aggregation and lung metastasis (44). Mutant rhodocytin and anti-podoplanin antibodies also reversed platelets promoting lung colonization in osteosarcoma (45). These results suggest that the interactions between CLEC-2 and PDPN are essential to TCIPA which is the protection of tumor cells in hematogenous metastasis (46). CLEC-2-PDPN axis becomes promising for antiplatelet and antitumor drugs. By blocking the CLEC-2-PDPN interaction through a polysaccharide-containing fraction from Artemisia argyi or cobalt hematoporphyrin (Co-HP), TCIPA and, subsequently, tumor metastasis can be inhibited (47, 48). Moreover, it has been speculated that CLEC-2 depletion did not show severe bleeding tendency, so that to some extent targeting platelet CLEC-2 is a safe treatment strategy (43, 46, 48). In addition, PDPN is also expressed in tumor stroma, including cancer-associated fibroblasts (CAFs) (49). PDPN-positive CAFs exhibit poor clinical outcomes in cancers of the lung (50), breast (51, 52), pancreas (53), and esophagus (54). PDPN-expressing CAFs are tumor-promoting by constructing immunosuppressive TME, which may be modulated by TGF-β production and CD204+ tumor-associated microphages infiltration (55, 56). PDPN-positive CAFs generate tracks through Rho-ROCK pathway to assist with cancer cell invasion in the extracellular matrix (57). Taken together, platelets and CAFs are likely to mediate tumor cell activities in tumor stroma jointly through CLEC-2 and PDPN, but the underlying mechanisms have not yet been studied.

Glycoprotein VI (GPVI), an immunoglobulin superfamily receptor, exerts multiple functions of platelets, particularly collagen-induced platelet activation. GPVI is demonstrated to promote colon and breast cancer cell metastasis by enhancing vascular permeability in response to its counter receptor galectin-3 on tumor cells. GPVI blockade using JAQ1 F(ab′)2 impairs platelet–tumor cell interactions and metastasis with only minor hemostatic side effects (58). Moreover, because GPVI is a critical regulator of vascular integrity in growing tumors, it becomes a promising target for antimetastatic therapies. Functional inhibition of platelet GPVI induces intra-tumoral bleeding and increases the efficacy of chemotherapeutic drugs (59). Although the involvement of GPVI in metastasis has rarely been studied (60–62), a few studies have reported that GPVI promotes experimental metastasis in Lewis lung carcinoma and melanoma models (34), and that GPVI inhibitor (revacept) and galectin-3 inhibitors prevent colon cancer metastasis in animal models (63). Very recently, an organ-on-chip platform called ovarian tumor microenvironment chip (OTME-Chip) was developed to verify how platelets extravasate through the endothelium into the tumor microenvironment, promoting tumor cell proliferation, metastasis, and chemoresistance. Again, the GPVI-galectin-3 interaction was identified to play a critical regulatory role in platelet–tumor interactions (64). Collectively, GPVI is exclusively expressed in platelets and megakaryocytes and hardly affects hemostasis as an antithrombotic target (65). Hence, targeting GPVI is a safe and effective antimetastatic strategy.

Reversible phenotypic changes in epithelial-mesenchymal transition of cancer cells involve loss of intercellular adhesion and enhanced motility and invasiveness. Platelets aid in the EMT process by enabling tumor cells to detach from the surrounding tissue of the primary tumor and intravasate into the circulation (66). TGF-β is one of the most important promoters in the EMT of tumor cells. Mesenchymal CTC clusters attached to platelets show high TGF-β signatures (67). It is pointed out that platelet-derived TGF-β could synergistically activate the TGF-β/Smad signaling and NF-κB pathways in tumor cells, and that abrogation of TGF-β signaling was sufficient to inhibit metastasis and EMT (68). Likewise, podoplanin-mediated EMT could be suppressed by a TGF-β neutralizing antibody (69). As an essential signaling molecule, TGF-β secreted from activated platelets is involved in multiple steps of metastasis cascades consisting of immunosurveillance, EMT, and invasiveness.

Interestingly, cancer cell-platelet interaction can be enhanced by the EMT process. Heat shock protein 47 (HSP47), a collagen-binding protein, has been found to be overexpressed in breast cancer and glioblastoma multiforme (GBM). Expression of HSP47 is correlated with cancer metastasis and tumor grade (70, 71). HSP47 is exposed on the surface of the platelet. It contributes to thrombosis and hemostasis, and its inhibitor, Col003, prevents the contact between platelet and collagen by inhibiting GPVI and mitogen-activated protein kinase (MAPK) signaling (72, 73). Furthermore, HSP47 expression in glioma vessels promotes glioma angiogenesis *via* HIF1α-VEGFR2 signaling (74). More importantly, the HSP47/collagen axis, which is crucial for cancer cell-platelet interaction, has been shown to promote cancer colonization and metastasis (75). During EMT, increased expression of HSP47 and collagen induces platelet recruitment and subsequently enhances CTCs clustering and extravasation. In the experiment, knockdown and silencing of HSP47, as well as the antibodies of collagen receptors in platelets (e.g., integrin α2β1 antibody and GPVI antibody (jAQ1)) are used to inhibit collagen-platelet interaction. However, targeting the interaction between HSP47 and collagen may depend on the small-molecule compound Col003 (76). Another study suggests that HSP47 could induce tumor cell stemness through the TGF-β pathway, implying a positive feedback loop between cancer cell–platelet interaction and EMT induction.

Among the three β1 integrin family members expressed on platelets, α2β1 and α6β1 are involved in direct interaction between tumor cells and platelets. According to recent research, integrin α2β1 is involved in platelet contact with the human breast cancer cell line MCF-7 and promotes EMT of tumor cells by activating the Wnt-β-catenin pathway. The activated Wnt-β-catenin pathway promotes the secretion of TGF-β1 in tumor cells and, in conjunction with TGF-β1/pSmad3 pathways, enhances the transcription of Snail and Slug, which are correlated with EMT (77). Both knockout of integrin α6 in the megakaryocytic lineage and pharmacological blocking of integrin α6 by GoH3 diminished experimental lung metastasis in mice. The study also identified ADAM9, which is expressed on tumor cells, as the counter receptor of α6β1. Their direct binding benefits platelet recruitment to CTCs and their subsequent activation and granule secretion. The interplay between platelet α6β1 and tumor ADAM9 facilitates tumor cell intravasation and extravasation, as evaluated by the trans-endothelial migration *in vitro* and *in vivo (* 78). The potential role of the platelet β1 integrin family in tumor metastasis needs more experimental evidence and clinical studies (79).

The establishment of metastasis is inseparable from tumor cell arrest, adhesion, and extravasation in a distant site. ATP plays a vital role in the disruption of endothelial junctions. ATP secreted by tumor cell-activated platelet dense granules was identified to act on P2Y2 receptor on endothelial cells and induce tumor cell extravasation and metastasis, especially tumor cell endothelial transmigration (80). Later studies also showed that ATP from the tumor microenvironment was involved in cell invasion or metastasis in breast, prostate, and gastric cancer *via* the P2Y2 receptor expressed in tumor cells (81–83).

Numerous studies have shown that platelet-derived lysophosphatidic acid (LPA) supports metastasis in breast cancer, ovarian cancer, osteosarcoma, and glioblastoma (84–86). The mechanism by which platelets contribute to cancer metastasis through LPA depends on autotaxin (ATX) which is overexpressed in multiple types of cancers and stored in α-granules of resting platelets. When tumor cells initiate platelet activation, ATX is released and mediates the production of LPA through its lysophospholipase D activity (87–89). Platelets are major sources of LPA, which regulates a variety of pleiotropic activities, such as proliferation, survival, motility, and autophagy (90). LPA receptor 1 (LPAR1) expressed in cancer cells is considered as an important response to LPA, and the absence of LPAR1 may affect vascular leak (84, 86, 88, 91). Tumor CD97, an adhesion G protein-coupled receptor (GPCR), stimulates platelet activation and mediates CD97-LPAR signaling (92). LPA is regarded as a significant bioactive molecule in tumor cell proliferation, invasion, and migration, especially trans-endothelial migration through vascular permeability. Therefore, the ATX-LPA signaling pathways give a new prospect, and LPAR1 and CD97 become promising therapeutic targets in the fight against cancer metastasis (93, 94). Interestingly, LPA and sphingosine 1-phosphate (S1P) also take part in angiogenesis by acting on endothelial cells (95).

### 2.3 Effects of TAPs on inducing or accessing the vasculature

The contribution of platelets to tumor angiogenesis has long been recognized. Multiple molecules secreted from platelet α-granules exhibit pro- and anti-angiogenic properties. However, the precise mechanisms of platelet granule secretion by different stimulations have not been fully understood. Pro-angiogenic factors contain vascular endothelial growth factor (VEGF), platelet-derived growth factor (PDGF), basic fibroblast growth factor (bFGF, also known as FGF2), epidermal growth factor (EGF), metalloproteinases (MMPs), etc. Anti-angiogenic factors include thrombospondin-1 (TSP-1), sphingosine 1 -phosphate (S1P), endostatin, platelet factor 4 (PF4/CXCL4), and so on. Previous studies showed that pro- and anti-angiogenic factors in separate platelet α-granules are released differently. Proteinase-activated receptors (PARs) counter-regulate the release of pro- and anti-angiogenic factors. To be specific, PAR1-activating peptide (PAR1-AP), ADP (via P2Y1/P2Y12), and GPVI-targeting collagen-related peptide induce the expressions of stromal cell-derived factor-1α (SDF-1α/CXCL12) and VEGF, but not endostatin. In contrast, PAR4 activation stimulates endostatin and platelet factor 4 release but suppresses the release of VEGF and SDF-1α (96–98). Similarly, adenosine diphosphate (ADP) and thromboxane A2 (TXA2) also counter-regulate platelet release and have the opposite effect on angiogenesis. ADP stimulates the release of VEGF and promotes the migration and formation of capillary structures. Conversely, the release of endostatin stimulated by TXA2 has an inhibitory effect (99).

Pro-angiogenic platelets, which are the drivers for the tumor’s angiogenic switch to break dormancy, can mediate the primary tumor’s effect on the systemic macroenvironment (26). Platelet α-granules are required for bone marrow-derived cell (BMDC) recruitment which is also important for the angiogenic process (100). Platelet-derived growth factor B (PDGFB) in platelet maintains tumor vessel integrity in the TME, which is dependent on the recruitment of pericytes (101). Chemotherapy targeting tumor vasculature also utilizes platelet biomimetic technology to achieve intra-tumoral vascular destruction (102).

### 2.4 Effects of TAPs on avoiding immune destruction

The most essential aspect of tumor immunosurveillance has been the engagement of natural killer (NK) cells through cytotoxicity and IFN-γ production (103–106). Platelets may directly protect tumor cells from NK tumor-lytic activity. Apart from surface shielding by platelet aggregates, numerous investigations showed that the interaction between platelet and fibrin (or fibrinogen) plays a crucial role in immune evasion (107, 108). Salih and colleagues explored various mechanisms, including the down-regulation of NKG2D on NK cells by platelet-derived TGF-β, platelet-derived MHC class I transfer onto the tumor cell surface, and forward signals from platelet-expressed glucocorticoid-induced TNF-related ligand (GITRL) to GITR on NK cells that result in the impaired anti-tumor reactivity of NK cells (109–111). These findings shed light on how platelets impact tumor-NK cell interactions. Overexpression of a hypoxia-inducible factor (HIF)-target gene in renal cancer cells may also enhance platelet binding, protecting cancer cells from NK cell-mediated cytotoxicity (112).

Platelets have also been proven to impact adaptive immunity. TGF-β and lactate are major immunomodulators of T cell activity from platelet releases. Platelet-intrinsic glycoprotein A repetitions predominant (GARP) that dominantly contributes to TGF-β activation subsequently suppresses CD4^+^ and CD8^+^ T cells. Platelet-specific deletion of GARP by gp96, a molecular chaperone of GARP and GPIb-IX-V, leads to enhanced tumor-specific T-cell immunity (113). The authors then discovered that platelet GARP cleavage, which is required for TGF-β maturation, is thrombin dependent. Thrombin inhibition reduces activated TGF-β and hence avoids immune tolerance (114). Therefore, the GARP-TGF-β axis may be the molecular mechanism of combination therapy of immunotherapy and anti-platelet agents in cancer (113, 115).

### 2.5 Effects of TAPs on tumor-promoting inflammation

Platelets participate in the inflammatory process, including interactions with leukocytes and endothelial cells, in addition to their vital functions in hemostasis and thrombosis. Platelets recruit and activate various immune cells and promote the secretion of cytokines through surface proteins and the release of pro-inflammatory or regulatory inflammatory factors (10). Granulocytes can be recruited to form early metastatic niches through CXCL5/7 chemokines derived from platelets (116). Immune responses to tumors, like infections, are large and complex, involving various cellular components and molecular pathways. The interactions between platelets and host immune cells potentially influence tumor-promoting inflammation (66).

Neutrophils, the main component of the innate immune system, respond to infection and are involved in tumors. Platelets–neutrophils cross-talk in infection and tumor is inseparable from neutrophil extracellular traps (NETs), a web-like structure composed of extracellular DNA decorated by histones and granular proteases. Platelets induce NET formation and bind to them, while NETs act as a scaffold for platelet adhesion and aggregation and facilitate platelet activation (117–119). A recent study demonstrated that surgical stress-activated platelets facilitated NETs-mediated capture of CTCs and synergized with the enhanced aggregation of platelet–tumor cells, contributing to distant metastasis *via* the Toll-like receptor 4 (TLR4)-ERK5-integrin GPIIb/IIIa axis (120). The high affinity between NETs and platelet-coated-CTCs created favorable conditions for tumor cell dissemination after surgical inflammation.

Furthermore, TLR4 is a mediator in microbial and sterile inflammation, as well as an important component of the lipopolysaccharide (LPS) receptor signaling complex expressed on platelets (121). The TLR4 pathway can activate platelets in response to high-mobility group box1 (HMGB1) released from dying tumor cells destroyed by NK cells and shear stress (122). These studies indicated that TLR4 on platelets is a potential target for reversing tumor-promoting inflammation and restrain tumor dissemination.

### 2.6 Effects of TAPs on deregulating cellular metabolism

Metabolic changes affect tumor growth, survival, and metastasis. Platelet metabolism is dependent on mitochondrial functions for energy requirements and even the lifespan of platelets (123). Intratumoral hypoxia triggers the release of chemokines and growth factors in platelet granules, while oxidative stress alters mitochondrial function of platelets (124). Platelets are metabolically active and can utilize glycolysis instead of oxidative phosphorylation (OXPHOS). Although no studies have shown that platelets can affect tumor cell mitochondria functions, platelets are thought to be biomarkers of mitochondrial dysfunction in cancers (125). Platelets can release mitochondria into circulation and transfer mitochondria to other cells, such as mesenchymal stem cells, under certain circumstances including wound healing, inflammation, and cancer, thereby building bridges for cell communications and promoting specific properties (126–129). In addition, tumor cells can concentrate mitochondria in the most active areas of the platelet–tumor cell process (130). As a result, we can speculate that platelet–tumor cell interplays may affect the mitochondria location and function of tumor cells. Platelets promote the aggressive phenotype of tumor cells, and tumor cells undergo metabolic reprogramming during EMT (131). The metabolic advantages of CTCs that platelets may provide in circulation include increased glucose uptake and lactate production (132).

## 3 Perspectives

**Figure 2:**
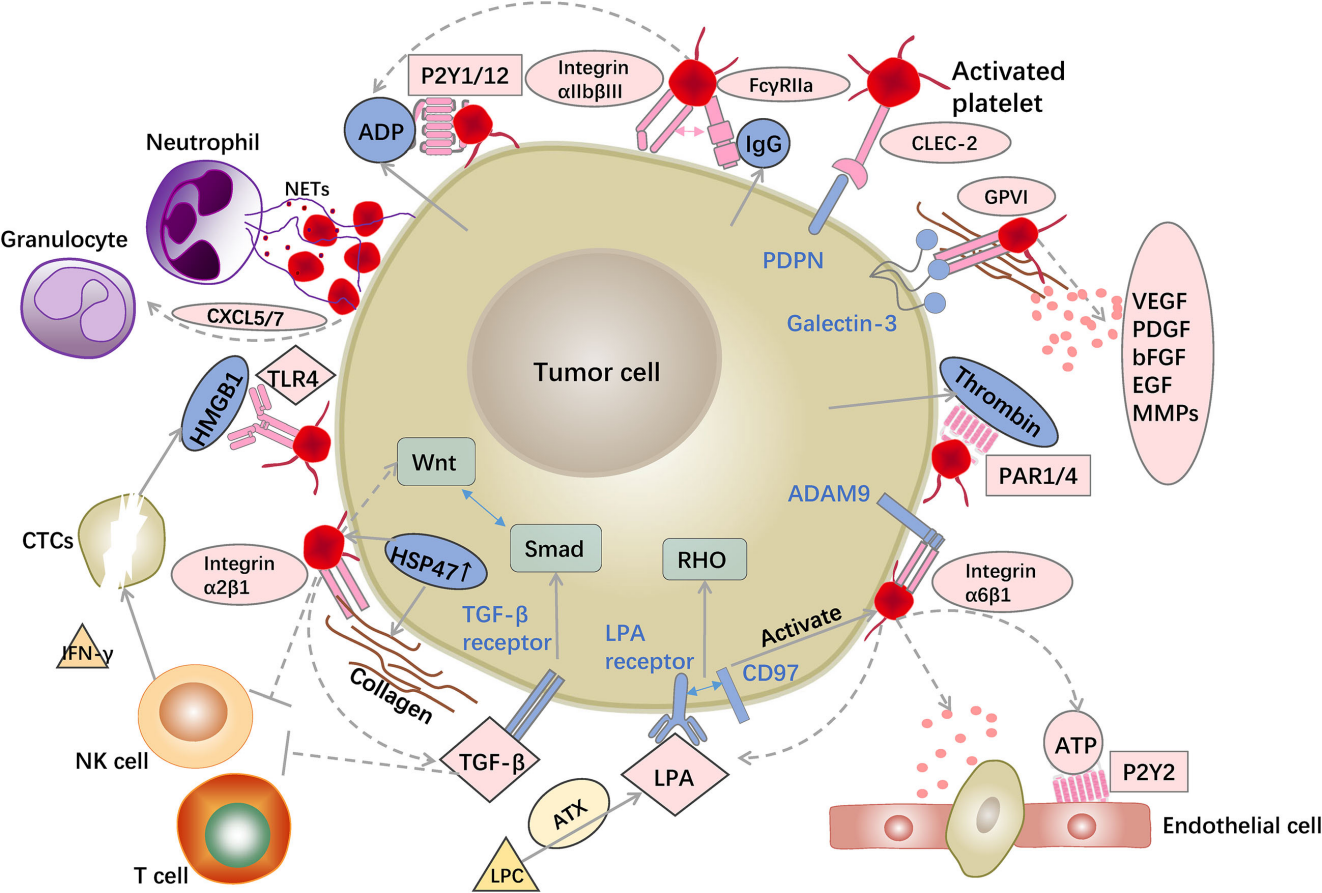
Examples of interactions between platelets and tumor cells. Platelet surface receptors and released 880 granules provide favorable conditions for tumor progression. Platelets recruit granulocytes via released factors and induce NET formation to bind to CTCs. Platelet surface receptors, such as FcγRIIa and CLEC-2, bind to tumor cells and promote platelet activation and aggregation. LPA and ATP released by platelets as well as platelet GPVI and integrin α6β1 aid tumor cell trans-endothelial migration. Platelets rich in TGF sources promote EMT of tumor cells. Platelets suppress NK and T cell activity, allowing tumor cells to survive in the bloodstream.

Given the recognition of the importance of TME, the roles of non-tumor cell components in tumor initiation, growth, and metastasis are increasingly studied. The roles of platelets in tumor biology are gradually recognized, and significant progress has been made in revealing the effects of platelets on cancer development. Herein, we reviewed the essential molecular mechanisms underlying tumor–platelet dynamic interactions from the perspectives of cancer hallmarks (**Figure 2**). Several platelet molecules can be potential anti-platelet targets and have been verified by experiments in animal models. However, platelet-targeted anti-tumor therapy has not formed a prospective treatment regimen. Profound knowledge of tumor–platelet interactions is critical for future research and novel therapeutic interventions.

Aspirin is widely used to prevent and treat cardiovascular and cerebrovascular diseases, and cyclooxygenase (COX) is an important target of aspirin. Clinical evidence has demonstrated that aspirin exerts the roles in chemoprevention of multiple cancers, such as colorectal, gastric, pancreatic, and breast cancers (133–136). However, numerous cohort studies found no effect or even increased risks of several types of cancers (137). The efficacy of aspirin for cancer prevention still needs more clinical trials to verify; furthermore, whether the combination of aspirin and other agents may be more effective than aspirin alone; whether patients can benefit from aspirin in the presence of potential side-effects or apply novel aspirin derivatives; whether aspirin can have a preventive effect in different population types or specific cancer subtypes; and whether the efficiency of aspirin may be dose-dependent, particularly at low doses. Many questions must be answered so that more individuals can benefit from aspirin use (138). Furthermore, the efficacy of aspirin for adjuvant cancer therapy has been validated (139). Aspirin inhibits platelet activation *via* COX-1 and epithelia and tumor cells *via* COX-2. Although aspirin and its metabolites have a variety of targets, platelet COX-1 may be the key mechanisms for the anticancer effects of low-dose aspirin (140). The anti-cancer effects of aspirin, which involve multiple cancer hallmarks, including tumor growth (141), metastasis (142, 143), angiogenesis (144, 145), immune evasion (113), are consistent with the platelet biology.

Drug delivery systems using nanoparticles are being developed as an alternative therapeutic route for chemotherapeutic drug transport that can minimize the side effects of chemotherapy in cancer patients (146). Nanoparticles modified with P-selectin-targeting agents contain the antiplatelet agent ticagrelor and the anti-inflammatory agent celecoxib. This nanoplatform focuses on tumor inflammation and tumor–platelet crosstalk and thereby various steps of metastatic cascades can be influenced. Platelet-blocking nanoparticles reduce the platelet-derived TGF-β and downregulate CXCL5 which decreases granulocytes recruitment. Inflammation inhibiting nanoparticles downregulate the MMPs and reverse the inflammatory status. The combination has synergetic effects on inhibiting EMT and interfering with inflammatory microenvironment (147). Based on the essential role of TGF-β in immune suppression and EMT, blocking the major source of TGF-β, which are the platelets, is an essential approach to prevent tumor metastasis. For this reason, Paclitaxel and a nitric oxide (NO) donor-modified albumin shell is constructed to release NO, which can block platelet functions including aggregation, adhesion, and coagulation. Platelet-induced EMT which includes morphological changes and protein markers can be weakened. Obviously, the levels of TGF-β derived from platelets can be downregulated so that tumor immunosuppression can be reversed. In other words, NO-inhibited platelet can be restrained from contacting with tumor cells which involves platelet adhesion around CTC, EMT, and distant metastasis (148). Platelet membrane-coated nanoparticles are also used as a biomimetic delivery approach of immunomodulator agents to inhibit tumor-promoting immune signaling which can make efficient immunotherapy come true (149). These studies exactly take advantage of platelet biological characteristics and abundant connections with cells in the tumor microenvironment. An important question is whether modified platelets carrying cancer therapeutics can be used to target established tumor masses in addition to tumor cells in the circulation. Better understanding of the dominant role of platelets in tumor progression has no doubt to be of guiding significance. Whether directly targeting the interaction between platelets and cancer cells by antiplatelet drugs or using platelets themselves as anticancer drug carriers, these still need to be verified in future clinical practice.

Both the old drug aspirin and the new nanotherapeutics affect tumor development by inhibiting platelet activity. In conclusion, platelet receptors, their binding partners, signaling proteins, and soluble molecules are all potential targets for anti-cancer drugs that target platelet–tumor cell interactions. Extensive experimental evidence for antiplatelet drugs supports the importance of platelets in tumor progression. It is hoped that traditional anti-platelet drugs and novel nanotechnology can be used in cancer treatment as a result of a comprehensive understanding of platelet physiology and the complex mechanisms underlying platelets in cancer biology.

## Author contributions

LC and FP wrote the original draft. LC and YC designed the figure. CZ collected information and revised this manuscript. LX reviewed this manuscript. YL, XC, and GH designed and revised this manuscript. All authors contributed to the article and approved the submitted version.

## Glossary

**Table:**

***ATP***	adenosine triphosphate
***ATX***	autotaxin
***bFGF/FGF2***	basic fibroblast growth factor
***BMDC***	bone marrow-derived cell
***CLEC-2***	C-type lectin-like receptor 2
***Co-HP***	cobalt hematoporphyrin
***COX***	cyclooxygenase
***CTC***	circulating tumor cells
***ECM***	extracellular matrix
***EGF***	epidermal growth factor
***EMT***	epithelial-mesenchymal transition
***FAK***	focal adhesion kinase
***FcγRIIa***	Fcγ receptor IIa
***GARP***	glycoprotein A repetitions predominant
***GBM***	glioblastoma multiforme
***GITRL***	glucocorticoid-induced TNF-related ligand
***GP***	glycoprotein
***GPCR***	G protein-coupled receptor
***GPVI***	glycoprotein VI
***HCC***	hepatocellular carcinoma
***HIF***	hypoxia-inducible factor
***HMGB1***	high-mobility group box1
***HSP47***	heat shock protein 47
***IgG***	immunoglobulin G
***ITAM***	immunoreceptor tyrosine-based activation motif
***LPA***	lysophosphatidic acid
***LPAR1***	LPA receptor 1
***LPS***	lipopolysaccharide
***MAPK***	mitogen-activated protein kinase
***miRNA***	micro-RNA
***MMPs***	metalloproteinases
***NAFLD***	non-alcoholic fatty liver disease
***NETs***	neutrophil extracellular traps
***NK***	natural killer
***NO***	nitric oxide
***OTME-Chip***	ovarian tumor microenvironment chip
***OXPHOS***	oxidative phosphorylation
***PAR***	proteinase-activated receptor
***PAR1-AP***	PAR1-activating peptide
***PDGF***	platelet-derived growth factor
***PDGFB***	platelet-derived growth factor B
***PDPN***	podoplanin
***PF4/CXCL4***	platelet factor 4
***PLR***	platelet to lymphocyte ratio
***PMP***	platelet-derived microparticles
***S1P***	sphingosine 1-phosphate
***SDF-1α/CXCL12***	stromal cell-derived factor-1α
***TAP***	tumor-associated platelets
***TCIPA***	tumor cell-induced platelet aggregation
***TCIPS***	tumor cell-induced platelet secretion
***TEP***	tumor-education platelets
***TGF-β***	transforming growth factor-β
***TLR4***	toll-like receptor 4
***TME***	tumor microenvironment
***TSP-1***	hrombospondin-1
***TXA2***	thromboxane A2
***VEGF***	vascular endothelial growth factor
***YFP***	yellow fluorescence protein
